# What’s needed to improve safety and quality of abortion care: reflections from WHO/HRP Multi-Country Study on Abortion across the sub-Saharan Africa and Latin America and Caribbean regions

**DOI:** 10.1136/bmjgh-2021-007226

**Published:** 2021-08-31

**Authors:** Hedieh Mehrtash, Caron Rahn Kim, Bela Ganatra, Özge Tuncalp

**Affiliations:** UNDP/UNFPA/UNICEF/WHO/World Bank Special Programme of Research, Development and ResearchTraining in Human Reproduction (HRP), Department of Sexual and Reproductive Health and Research, World Health Organization, Geneva, Switzerland

**Keywords:** obstetrics, public health, cross-sectional survey

**Summary box**

- Complications as a result of unsafe abortion are an important and preventable cause of maternal mortality and morbidity.
- Based on indicators present at the time of facility admission, abortion-related complications were classified into five hierarchical and mutually exclusive categories based on severity: (1) severe maternal outcomes consists of mortality and near miss (3) potentially life-threatening complications, (4) moderate complications and (5) mild complications.
- Across sub-Saharan Africa and Latin America and Caribbean regions, our findings illustrate that the majority of complications were moderate and mild complications, and marginally more severe complications in sub-Saharan Africa.
- Women’s experiences of abortion care across both regions underlined the need for effective communication and emotional support including reducing anxiety and stress during examinations.
- A multi-pronged approach including self-care, clinical care, task sharing, human rights and enabling legal environment is needed to deliver high-quality abortion and postabortion care including access to contraceptives.

## Introduction

In the advent of safe methods, access to information and trained providers, abortion has become a very safe procedure; however, unsafe abortions continue to persist in many parts of the world. Unsafe abortions account for half of all abortions globally, with the majority occurring in sub-Saharan Africa and Latin America and Caribbean (LAC).^1^ Severe abortion-related complications arise from least safe abortions.^1^ Between 2008 and 2013, it was estimated that approximately 10% of maternal deaths are attributable to abortion-related causes in sub-Saharan Africa and LAC^2^; however, studies exploring abortion morbidity and mortality including management of these complications have been limited or varied in estimations of the complications limiting comparability as there has been a lack of standard definitions, identification criteria and measurement tools.

## WHO/Human Reproduction Programme (HRP) Multi-Country Study on Abortion

Since the early 2000s, WHO/HRP has been conducting a series of WHO Multi-Country Studies (MCSs) on maternal and newborn health across multiple countries and health facilities globally.^3–5^ Using the original WHO MCS methodology and network,^6^ the WHO Multi-Country Study on Abortion (MCS-A)^7^ study aimed at measuring the prevalence and management of abortion-related complications across health facilities in LAC and sub-Saharan Africa. One of the key contributions of this MCS was to use standardised definitions for severity of complications using WHO criteria on near miss and potentially life-threatening conditions.^8^ Given the growing interest around women’s experiences of care, the study also adapted WHO’s quality of care framework on maternal and newborn health to explore women’s self-reported experiences of postabortion care (including contraception method of choice) as pertained to respect and dignity, effective communication and support.^9^

**Figure 1:**
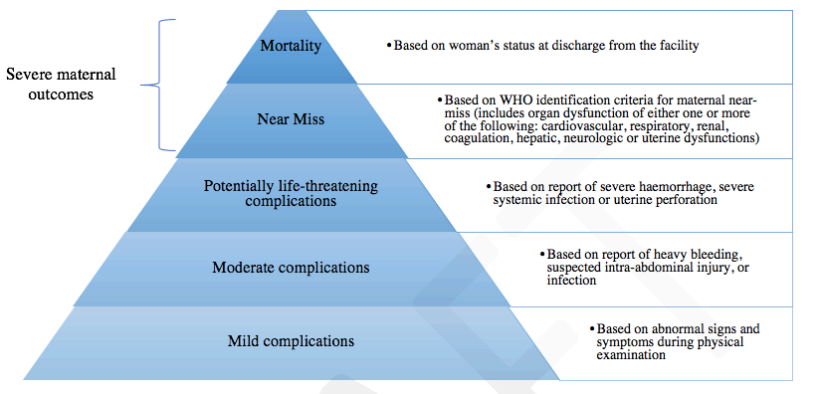
WHO/Human Reproduction Programme Multi-Country Study on Abortion severity of abortion-related complications.

Data on women seeking care for abortion-related complications, including ectopic and molar pregnancies at the time of facility admission were abstracted from medical records. Abortion-related complications were classified into five hierarchical and mutually exclusive categories based on severity^1^: deaths,^2^ near miss,^3^ potentially life-threatening complications,^4^ moderate complications and^5^ mild complications (figure 1). In addition to the original methodology, an Audio Computer-Assisted Self-Interviewing (ACASI), a system well-suited for collecting data confidentially on sensitive topics such as abortion were included to document women’s experiences of care.^10 11^

As a result, the WHO MCS-A was conducted in 17 countries across sub-Saharan Africa and the LAC regions.^12 13^ In Africa, the study was conducted in 211 facilities across Benin, Burkina Faso, Chad, Democratic Republic of Congo, Ghana, Kenya, Malawi, Mozambique, Niger, Nigeria and Uganda. In LAC, the study was conducted in 70 facilities across Argentina, Brazil, Bolivia, Dominican Republic, El Salvador and Peru.

## Severity and management of abortion-related complications

**Figure 2:**
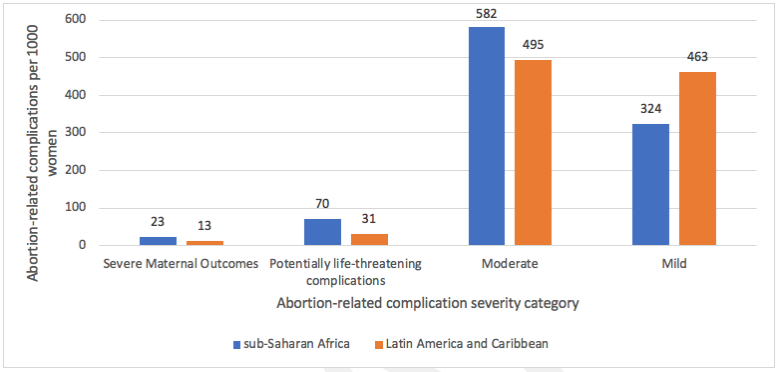
Abortion-related complications across sub-Saharan Africa and Latin America and Caribbean regions.

Across both regions, our findings illustrate that the majority of complications were moderate and mild complications with marginally more severe complications identified in sub-Saharan Africa (figure 2). This is in line with abortion safety estimates where sub-Saharan Africa is estimated to have the highest proportion of abortions that are conducted under least safe conditions leading to severe complications.^1^

In sub-Saharan Africa and LAC, women who were single, presenting ≥13 weeks of gestational age and where expulsion of products of conception occurred prior to arrival to facility were more likely to experience severe abortion-related complications. Abortion remains a stigmatised issue limiting equitable access to care for women based on their socioeconomic characteristics, such as marital status, contributing to severe complications. In restrictive settings, not only are services for safe abortion limited but vague abortion laws and policies, lack of access to accurate information, medication, equipment and limitations on the provider types who can provide abortion care, are contributing factors that may lead women to delay seeking care for complications if they do occur.^14^

In terms of management, manual vacuum aspiration and misoprostol were most commonly used to manage abortion-related complications. In both regions, the use of dilation and curettage was still used. Another striking finding from our study is the ample use of antibiotics to manage abortion-related complications highlighting the need to regulate and promote appropriate use of antibiotics and combat antibiotic resistance.^15^ Global efforts must focus on transitioning to evidence-based methods both for provision of abortion and management of complications.

Via ACASI, both in in sub-Saharan Africa and LAC, of those who responded that method was used to induce their abortion, one in two women reported using misoprostol. Used correctly, misoprostol (used in combination with mifepristone) or by itself is a safe and effective method recommended in WHO guidelines.^16–18^ Provision of safe abortion care requires effective adoption and implementation of WHO recommendations. Strengthening these efforts will be essential to those involved in quality abortion care, in particular the women, providers and policy makers.

## Women’s experiences of postabortion care

While there is room for improvement needed on the provision of postabortion care and management, women’s experiences of care across both regions underlined the need for emotional support including reducing anxiety and stress during examinations, and effective communication such as having questions answered during the time of care. These findings warrant further research around improving quality of care, especially focusing on experience of care during this time. To explore some of these issues in the study database, the study team is currently working on a set of secondary analyses to be published in 2021. While more work is needed on understanding and improving women’s experiences of care, it will be pertinent to tackle stigma surrounding abortion globally. Evidence on various approaches to reduce abortion stigma have clearly shown that it hinders access at all levels of level care.^19^ Future programmes must consider how stigma can affect delays to care, access to accurate information, and available social and financial support, all of which have economic and health implications. It will be important to use new approaches to reach women that are not reaching facility-level care. WHO is currently exploring network-based methods^20^ to better understand how women’s social networks can deter or refer her to reach safe abortion care services.

## Conclusion

Unsafe abortion is a serious area of concern in sub-Saharan African and LAC countries resulting in significant morbidity for women and burden on health systems. It will be important to use a standardised approach to quantify abortion-related complications and incorporate women’s experiences of abortion care to improve postabortion care services. It is critical to decrease the risk of severe abortion morbidity by using innovative outreach efforts to provide women with appropriate information and support to reduce delays in care-seeking. Moving forward it is important to incorporate a multi-pronged approach including self-care, clinical care, task sharing, human rights and enabling legal environment to deliver high-quality abortion and postabortion care including access to contraceptives.

## Data Availability

Data available upon request. The data used for this analysis might be made available upon reasonable request, in accordance with the WHO/HRP MCS-A Research Group data sharing policy and WHO Policy of Data Use and Data Sharing. For further information, contact srhmph@who.int and srhpua@who.int.
